# Electrophysiological evaluation of the effect of peptide toxins on voltage-gated ion channels: a scoping review on theoretical and methodological aspects with focus on the Central and South American experience

**DOI:** 10.1590/1678-9199-JVATITD-2023-0048

**Published:** 2024-09-02

**Authors:** Jessica Rojas-Palomino, Alejandro Gómez-Restrepo, Cristian Salinas-Restrepo, César Segura, Marco A. Giraldo, Juan C. Calderón

**Affiliations:** 1 Biophysics Group, Institute of Physics, University of Antioquia, Medellín, Colombia.; 2 Physiology and Biochemistry Research Group -PHYSIS, Faculty of Medicine, University of Antioquia, Medellín, Colombia.; 3 Toxinology, Therapeutic and Food Alternatives Research Group, Faculty of Pharmaceutical and Food Sciences, University of Antioquia, Medellín, Colombia.; 4 Malaria Group, Faculty of Medicine, University of Antioquia, Medellín, Colombia.

**Keywords:** Patch-clamp techniques, Cellular model, Excitable cell, Venoms, Ionic current, Calcium channels, Potassium channels

## Abstract

The effect of peptide toxins on voltage-gated ion channels can be reliably assessed using electrophysiological assays, such as the patch-clamp technique. However, much of the toxinological research done in Central and South America aims at purifying and characterizing biochemical properties of the toxins of vegetal or animal origin, lacking electrophysiological approaches. This may happen due to technical and infrastructure limitations or because researchers are unfamiliar with the techniques and cellular models that can be used to gain information about the effect of a molecule on ion channels. Given the potential interest of many research groups in the highly biodiverse region of Central and South America, we reviewed the most relevant conceptual and methodological developments required to implement the evaluation of the effect of peptide toxins on mammalian voltage-gated ion channels using patch-clamp. For that, we searched MEDLINE/PubMed and SciELO databases with different combinations of these descriptors: “electrophysiology”, “patch-clamp techniques”, “Ca^2+^ channels”, “K^+^ channels”, “cnidarian venoms”, “cone snail venoms”, “scorpion venoms”, “spider venoms”, “snake venoms”, “cardiac myocytes”, “dorsal root ganglia”, and summarized the literature as a scoping review. First, we present the basics and recent advances in mammalian voltage-gated ion channel’s structure and function and update the most important animal sources of channel-modulating toxins (e.g. cnidarian and cone snails, scorpions, spiders, and snakes), highlighting the properties of toxins electrophysiologically characterized in Central and South America. Finally, we describe the local experience in implementing the patch-clamp technique using two models of excitable cells, as well as the participation in characterizing new modulators of ion channels derived from the venom of a local spider, a toxins’ source less studied with electrophysiological techniques. Fostering the implementation of electrophysiological methods in more laboratories in the region will strengthen our capabilities in many fields, such as toxinology, toxicology, pharmacology, natural products, biophysics, biomedicine, and bioengineering.

## Background

Cellular electrophysiology allows for the study of the electrical properties of mammalian cells, either non-excitable or excitable. These properties arise from the interplay of several mechanisms: the specific chemical composition of the biological membranes, the differential distribution of ions such as Ca^2+^, K^+^, Na^+^, and Cl^-^ across the cell membranes, and the presence of ion channels in those membranes. A reliable measure of the function of such ion channels became possible only until the development of the patch-clamp technique in the late 1970s and early 1980s by Neher and Sakmann [1-3].

On the other hand, an important number of laboratories were created during the last two decades in Central and South America focused on studies of natural products of vegetal and animal origin. However, even when natural products are gold mines to find regulators on ion channels, most studies in the field lacked complementary electrophysiological approaches. In a few cases, the new molecules were characterized in North America or Europe. Besides infrastructure limitations, this likely happens because researchers are unfamiliar with the scope of the electrophysiological techniques and their suitable cellular models.

Given the potential interest of many research groups in the highly biodiverse region that constitutes Central and South America, here we review the most relevant conceptual and methodological developments of the synergy produced by the study of natural products with an electrophysiological approach. Classical and novel information about the voltage-gated ion channel’s structure and function is presented, as well as an update of the most important mammalian voltage-gated ion channel-modulating toxins from animal sources (e.g. cnidarian and cone snails, scorpions, spiders, and snakes), highlighting the channel-interacting toxins described in Central and South America. Then, we explain the local experience of implementing electrophysiological techniques in experimental models of excitable cells, and the participation in screening natural products from animals (venoms, fractions, or toxins) and characterizing new modulators of ion channels. For the latter, we present an instructive, innovative study case of a toxin from a local spider, something uncommon in a region that has focused on scorpion toxins. These developments are expected to strengthen the capabilities of the region in fields like pharmacology, toxicology, toxinology, natural products, biophysics, biomedicine, and bioengineering.

## Methods

This scoping review summarizes conceptual and methodological relevant information retrieved from MEDLINE/PubMed and SciELO databases. The search was performed with different combinations of the following descriptors: “electrophysiology”, “patch-clamp techniques”, “Ca^2+^ channels”, “K^+^ channels”, “cnidarian venoms”, “cone snail venoms”, “scorpion venoms”, “spider venoms”, “snake venoms”, “cardiac myocytes”, and “dorsal root ganglia”, with no language or year constraints. For the sake of clarity and conciseness, because of the lack of Central and South American literature on other channels, and real future potential practical applications in laboratories of the region, we did not extend the review to other channels or cellular experimental models. 

The titles of all original papers retrieved until September 2023 were screened independently by two researchers and deemed eligible if they seemed to present novel experimental information on the structure of voltage-gated Ca^2+^ and K^+^ channels, described the effect of one or more peptide toxins on mammalian voltage-gated Ca^2+^ and K^+^ channels using electrophysiological techniques or detailed a patch-clamp protocol for studying peptide toxins on cardiac myocytes or dorsal root ganglia. A further refinement was then performed to identify non-redundant examples of the most important mammalian voltage-gated interacting toxins from cnidarian and cone snails, scorpion, spider, and snake sources, as well as the literature with key participation of research laboratories from Central or South America, between 1996 and 2023.

**Figure 1:**
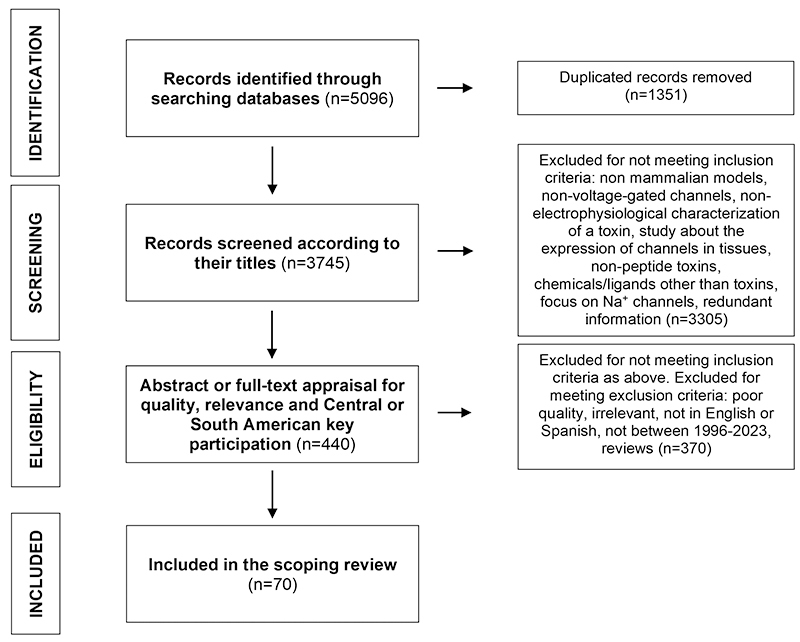
Flow diagram with the process of selection of the sources of evidence.

The quality of the papers was judged according to the quality of writing and reporting of their results, use of appropriate statistical approaches, number of experiments performed (including controls), and coherence among the objectives, methods used and conclusions reached. Finally, a few documents in languages other than English or Spanish were removed. The reporting of results of the present work follows the Preferred Reporting Items for Systematic Reviews and Meta-Analyses Extension for Scoping Reviews (PRISMA-ScR) guidelines [4]. Figure 1 presents a flow diagram with the process of selection of the sources of evidence.

## Conceptual aspects

### Ca^2+^ and K^+^ channels in excitable cells

Excitable cells can develop and conduct action potentials (AP), i.e., rapid changes in the potential difference across the membrane, as a response to a stimulus (e.g., mechanical, electrical or chemical). This property mediates the communication between cells and participates in multiple regulatory processes involving intracellular metabolism, signal transduction, sensing the environment, learning, gene expression, secretion of hormones, muscle contraction, and protein degradation, among others [5]. The main molecular mechanism underlying this property is the transport of ions in a concerted fashion through voltage-gated ion channels [6]. These channels are enriched in the membrane of the excitable cells, such as neurons and muscle cells, but are also present in some cells with negative resting membrane potentials, but which do not fire AP, such as the immune cells [5,7].

Ion channels have one of two typical structures, either a large, monomeric α-subunit or a polymeric α-subunit composed of smaller subunits. Voltage-gated Ca^2+^ channels represent the first structure and voltage-gated K^+^ channels are the prototype of the second.

*Voltage-gated Ca* ^*2+*^ channels (Ca_*v*_ )

**Figure 2:**
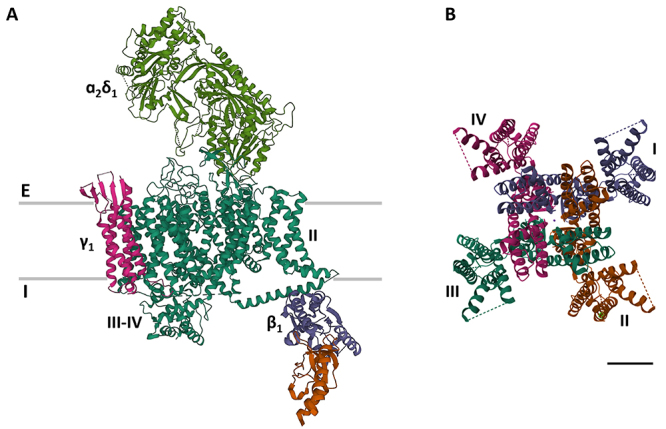
Structure of voltage-gated Ca^2+^ and K^+^ channels. **(A)** Lateral view of the structure of the mammalian voltage-gated Ca_v_1.1 complex. Gray lines represent the exterior (E) and interior (I) boundaries of the cellular membrane. II and III-IV indicate domains of the homologous α-subunit (green). Other subunits (α_2_δ_1_, γ_1_, β_1_) are also indicated. **(B)** Top view of the voltage-gated K_v_1.3 channel, showing the pore in the center. I to IV indicate the monomeric α-subunits. Structures taken from the Protein Data Bank (rscb.org), IDs: 5GJV and 7SSX, for Ca_v_1.1 and K_v_1.3, respectively. Calibration bar: 2 nm.

Changes in the cell membrane potential (*V*_*m*_ ) activate Ca_v_, leading to a Ca^2+^ influx, in turn involved in the regulation of many physiological processes, such as the release of neurotransmitters in neurons, the contraction of cardiomyocytes, or the sarcoplasmic Ca^2+^ release in skeletal muscle [8,9]. These channels are composed of one α-subunit, and several accessory subunits, as recently confirmed by cryoelectron microscopy reconstructions done with a resolution below 3.6 Å [10,11]. The α-subunit (Figure 2 A ) consists of a monomer with four homologous domains (DI-DIV) and is the principal component of the channels because it has the transmembrane pore domain (PD) and the voltage sensing domain (VSD) [9]. Each homologous domain contains six transmembrane α-helices (S1-S6) with a membrane-reentrant loop between them and cytoplasmic regions at the N and C terminal ends. The S5 and S6 helices constitute the PD and the S4 is the voltage-sensing helix of the VSD (S1-S4). The pore creates a permeation path for Ca^2+^ and crosses from the negatively charged dome region, which faces the extracellular space, to the intracellular space. The narrowest part of this permeation path is known as the selectivity filter (SF). The external region of the SF contains a couple of glutamate residues in each domain, which are required for Ca^2+^ ions selectivity. Each S6 forms the internal region of the SF from DI-DIV and constitutes a binding site for some Ca^2+^ antagonists, such as diltiazem and verapamil [10-12]. The S4 consists of repeated motifs of positively charged residues arginine and lysine, followed by two hydrophobic residues. During depolarization, the S4 moves towards the extracellular space, producing a conformational change in the PD and allowing the passage of ions. The inactivation gate is the loop linker between DIII-S6 and DIV-S1. Accessory subunits β, γ and α_2_δ modify biophysical and pharmacological properties besides influencing the abundance and trafficking of Ca_v_ channels [9,13] (Figure 2 A ).

Three major subfamilies of Ca_v_, Ca_v_1, Ca_v_2, and Ca_v_3 [9,14,15], underlie the existence of six different types of Ca^2+^ currents (L-, N-, P-, Q-, R-, and T-type). Ca_v_1.1, Ca_v_1.2, Ca_v_1.3, and Ca_v_1.4 have been cloned for the L-type Ca^2+^ current (I_CaL_, L stands for large conductance and long openings). They are activated from about -30 mV and are highly sensitive to dihydropyridines (DHP) [16]. Ca_v_1.1 is expressed in the skeletal muscle, while Ca_v_1.2 and Ca_v_1.3 are in the heart and neurons. Ca_v_1.4 expression is major in the retina, spinal cord, and immune cells. Ca_v_2.1 (P/Q-type, I_CaP/Q_), Ca_v_2.2 (N-type, I_CaN_), and Ca_v_2.3 (R-type, I_CaR_) are expressed in neurons of the cerebellum, brain, and peripheral nervous system [12]. Three genes *(CACNA1G, CACNA1H*, *CACNA1I*) encode for channels Ca_v_3.1, Ca_v_3.2, and Ca_v_3.3, responsible for the T-type currents (I_CaT,_ tiny currents, almost insensible to DHP, that activate at more negative potentials, i.e. −70 to −40 mV, and inactivate fast) [16]. Ca_v_3.2 is expressed in embryonic heart tissue. In adults, Ca_v_3.1 expression is higher than Ca_v_3.2 [16]. Given its expression, these channels are targets for the treatment of pain, stroke, epilepsy, migraine, and hypertension [17].

*Voltage-gated K* ^*+*^ channels (K_*v*_ )

K_v_ is by far the largest and most diverse family of ion channels and underlies a large number of K^+^ currents (I_K_) with different kinetics. They are largely responsible for the repolarization phase of the AP and are important in signal transduction, immunity, and blood pressure [7,18-20]. Structurally, their α-subunit is composed of individual monomeric, not necessarily identical, subunits (DI to DIV), which assemble within the membrane. Each monomeric domain contributes with the S5 and S6 to build a pore (PD) down their center (Figure 2 B ), while S4 works as the voltage-sensing helix of the VSD. The SF in the PD is formed by oxygens from the lateral chains of threonine (T) and the residues glycine (G) and tyrosine (Y), in a TXGYG sequence, where X represents a variable residue (usually valine (V) or isoleucine (I)). Like in the Ca_v_, the VSD is equipped with about five positively charged amino acids (arginine or lysine) at every third position. Depending on the direction and magnitude of the electric field, the VSD conformation changes, leading to the pore’s opening [21-25]. The mechanism of K^+^ conduction in its SF has been elucidated by molecular dynamics and the knowledge of its structure through X-ray diffraction of the KcsA channel [25,26]. 

In mammalian genomes, the α-subunits of the K_v_ channels are encoded by 40 different genes, classified into 12 distinct subfamilies. *Shakers* (K_v_1.1 to K_v_1.8), are expressed in the nervous system, heart, skeletal and smooth muscle, pancreas, lung, placenta, kidney, retina, colon, and T cells. *Shabs* (K_v_2.1 and K_v_2.2), are present in the brain, heart, neurons, and smooth muscle. *Shaws* (K_v_3.1 to K_v_3.4), are located in skeletal muscle, pancreas, liver, and lymphocytes. *Shals* (K_v_4.1 to K_v_4.3), are present in the brain, heart, smooth muscle, and neurons. *KCNQ* (K_v_7.1 to K_v_7.5) family is expressed in the nervous system, heart, pancreas, ear, kidney, lung, colon, placenta, and skeletal muscle. Ether-a-go-go related gene ‒*ERG*‒ (K_v_10.1, K_v_10.2, K_v_11.1, K_v_11.3, K_v_12.1, K_v_12.3) channels are generally found in the central nervous system and heart [15,18,22].

A fascinating characteristic of the K_v_ channels is their functional diversity, as manifested in a very variable spectrum of I_K_ kinetics. This can be explained in part because of the existence of structural differences among the large number of α-subunit isoforms mentioned above. However, another explanation includes the presence of a sizeable number of families and isoforms of auxiliary subunits, which regulate the intracellular trafficking, membrane location, voltage dependency, and inactivation of the K_v_ channels. These auxiliary subunits include K_v_β, KChAP (K_v_ channel-associated protein), KChIP (K_v_ channel-interacting protein), KCNE (K^+^ voltage-gated channel subfamily E regulatory subunit), and DPPX (Dipeptidyl-aminopeptidase-like protein 6) [5,23,27,28]. A complex relationship between α- and auxiliary subunits creates many possible functional channels with one α-subunit and one or more auxiliary subunits, as it has been brightly demonstrated for instance for the K_v_7.1/KCNE1/KCNE3, K_v_7.1/KCNE1/KCNE4, and K_v_7.1/KCNE3/KCNE4 heteromeric complexes [29]. Moreover, monomers of α-subunits of some isoforms may ensemble with monomers of other α-subunit isoforms in heteromeric functional channels which may have membrane locations and biophysical properties different from their mother isoforms, as it has been shown for K_v_1.4/K_v_1.6/KCNE channels in experimental models of channels expression, for K_v_7.1/K_v_7.5 in smooth muscle cells and for K_v_4.2/K_v_4.3/KChIP2 heteromeric complexes in murine cardiomyocytes [30-32].

K_v_ and Ca_v_ have essentially the same states, controlled by the transmembrane potential [5,33]. The opening of the gate (activation) is triggered by the membrane depolarization that energetically favors a continuous water-filled pathway where channels selectively discriminate which ions pass across the membrane. Subsequently, and even when the depolarization stimulus persists, the channels become inactivated. It means that the S4 helix stays in the “up” configuration, while the pore is still in the *open* conformation, but in a nonconducting state. Upon membrane repolarization, i.e., when the stimulus is removed, the channels are *deactivated*, acquiring the *closed* configuration [34].

Voltage-gated ion channels are one of the best-studied types of transmembrane proteins, in part because of the availability of a powerful tool: patch-clamp. This electrophysiological technique has led to a wealth of knowledge about the nature of the biophysical and physiological mechanisms of channel gating. Besides, patch-clamp allows to study the channels as targets of natural or synthetic pharmacological regulators. 

### The patch-clamp technique

In the late 1970s, recordings of channel currents flowing across the membrane were performed for the first time by Sakmann and Neher [2]. While improving the method, the possible applications of the patch-clamp technique to study ion channels in many cell types were clear [1]. Both researchers were thus awarded the Nobel Prize in Physiology or Medicine in 1991 [35]. Currently, patch-clamp is the gold standard for studying ion channel currents, because it offers quantitative information about the relationship between the transmembrane potential and the ion movement in living cells [36]. 

A conventional patch-clamp setup requires a system for mechanical and electromagnetic isolation, a magnifying system for visualization of the preparation, a stage and an experimental chamber suitable for the cellular models, equipment to fabricate the micropipettes, a micromanipulator, the electrical amplifier system, a digitizer and software, and hardware adequate for signal acquisition and processing (see section “I_Ca_ and I_K_ studies”).

The technique starts by establishing physical contact with a cell membrane using a glass micropipette filled with an electrolytic solution and a recording electrode (then becoming a microelectrode). The solution inside the micropipette typically resembles the composition of the intracellular milieu. Once the contact is established, a gentle suction is applied through the microelectrode, and a part of the membrane is suctioned into the pipette, with the subsequent increase of the electrical resistance. If done properly, a mechanically stable, high-impedance ‘gigaseal’ (> 1 GΩ) is established [1]. The gigaseal is key to increasing the signal-to-noise ratio (SNR) of the current signal. Starting from the gigaseal formation (cell-attached configuration), different configurations can be achieved [1,36-39].

If the patch membrane is ruptured, the whole-cell configuration is obtained. This configuration is particularly important and the most frequently used because direct contact between the cell’s cytoplasm and the micropipette’s internal solution is created. It allows for the study of the total (so-called macroscopic) membrane ionic currents and the AP, depending on the recording mode, voltage-clamp, or current-clamp, respectively [36-40]. A second, reference (also known as ground) electrode is placed in the bath solution (external to the cells) to complete the circuit. The composition of the bath solution resembles that of the extracellular medium.

The circuitry for voltage-clamping the cell membrane is particularly important for the success of the experiments. In a typical whole-cell, voltage-clamp experiment, a command potential (V_c_) generated by the amplifier system is imposed for a defined period (usually between 100 ms and 2 s) to the microelectrode, which is in turn expected to be transferred to the cell membrane so that V_m_ = V_c_. To do so, the device is equipped with a negative feedback function that rapidly and continuously compares V_m_ with V_c_ and if different, injects current. As long as the membrane seal resistance remains high, V_c_ and V_m_ become virtually identical in a few microseconds. This procedure is repeated to sequentially clamp V_m_ at different voltages in steps which are typically of 10 mV, in the so-called voltage-clamp protocol [36-39].

As stated, V_m_ is responsible for the conformational change of the VSD of the studied channels [5], therefore the different voltage-clamp steps lead to the graded opening of the channels. The consequent reduction of the membrane resistance to the ions creates a flow of ions (i.e., movement of charges), measurable as an ionic current, I_x_. The total current, I_t_, flowing through the membrane is the sum of all the ionic currents I_x_ and the capacitive current, I_C_:

$$I_{t}=I_{C}+\sum I_{x}$$ (1)

I_C_ depends on the membrane capacitance (C_m_):

$$C_{m}=\varepsilon \frac{A}{d}$$ (2)

Where ԑ is the dielectric constant, A is the membrane area, which changes according to the cell size, and d is the membrane thickness, which can be considered constant. 

Since the current flowing through the channels may change V_m_, an extra amount of current is then injected by the system through the microelectrode so V_m_ does not diverge from V_c_ during the voltage step. The magnitude of the extra current injected serves as a measure of the current flowing through the microelectrode and thus through the ion channels present in the membrane so that I_x_ is reported by the patch-clamp device for each voltage step [36-39]. The magnitude of I_x_ depends on the conductance of the ion (𝑔_x_) and the difference between V_m_ and the reversal potential of the ion, V_x_:

$$I_{x}=g_{x}(V_{m}-V_{x})$$ (3)

The values of I_x_ are usually normalized to the C_m_ (also given by the device) to correct for cell size and the number of functional α-subunits in the cell membrane [38]. The units of this current density are thus A/F (Ampere/Farad), but generally presented as pA/pF (picoAmpere/picoFarad), which will be identical in value.

Once in the open state, each channel displays a unitary single-channel conductance, 𝑔. The macroscopic conductance, G, of the channels in a membrane, is the ratio of I_x_ to the external voltage imposed at each voltage step of the current to voltage (I-V) plot [37,41]. Thus, G is the product of the total number of opened channels. The channel-open probability is the normalized macroscopic conductance to its maximum value (G/G_max_) [37,42].

Reducing electronic noise in cell electrophysiology is crucial. In patch-clamp, the largest individual noise may dominate total noise. This is a consequence of the fact that most of the noises in this technique are uncorrelated, which allows the expression of the total root mean square (RMS) noise as the square root of the sum of the individual squared RMS noise sources $\left(e_{T}^{2}=e_{1}^{2}+e_{2}^{2}+e_{3}^{2}\ldots \right)$ [43]. Background noise arises from a variety of sources: Johnson’s noise of the membrane-seal combination, the “shot noise” from ions crossing the membrane, intrinsic noise in the pipette, and the noise in the current-to-voltage converter and capacitance’s transients [1,37]. Noise can be largely controlled by grounding the setup, using a system for electromagnetic isolation, avoiding high temperatures during experimentation, ensuring high-resistance seals, and low-pass filtering the signals. Series resistance compensation (Rs), capacitance transient cancellation, and whole-cell compensations have been developed to reduce some types of noise sources and improve the quality of voltage-clamping so that the voltage control is optimal and the SNR can be the largest possible [1,43-45]. 

Besides recording macroscopic currents, the patch-clamp technique permits the study of the effects of toxins on single-channel kinetics. This can be accomplished with cell-attached, inside-out, or outside-out configurations [1,36-39,46]. Cell-attached have the advantage of allowing to perform single-channel recordings without exchanging the content of the microelectrode with the cytosol, hence avoiding any biochemical disturbances in the intracellular milieu. These high-resistance approaches permit the investigation of the gating properties of an individual or a few channels (usually up to two or three) under the effect, for instance, of different ligands. The signals can be analyzed for open and closed time durations and offer information about the conductance of the channels.

It is worth mentioning that manual and automated patch-clamp are complementary techniques. The latter has recently emerged as a high-throughput screening approach to study the effect of toxins on different ion channel isoforms [47].

Two-electrodes voltage-clamp is another electrophysiological method in which one electrode is focused on measuring the V_m_ and the other is destined to inject current. This improves the voltage control mainly when performing experiments in large cells, more reliably tracking the open and close kinetics of the channels when evaluating the effect of toxins [48-51].

Whatever the configuration or the number of electrodes used, obtaining a good signal depends on the quality and resolution of the equipment used in the setup and the technical skills of the researchers. In any case, patch-clamp is an invaluable tool in studying ion channels [1,37]. In our laboratory (see section “I_Ca_ and I_K_ studies”), the cutting-edge electronic components possess sufficient resolution to record currents for long-term experiments, with low noise.

### Why do we study animal toxins with electrophysiological techniques?

*General concepts*

Animal venoms are mixtures of different types of biomolecules that include proteins, peptides, amino acids, neurotransmitters, and salts, which alter physiological processes in the inoculated animal. Toxins are the main active compounds of venoms, which are widespread among invertebrate and vertebrate phyla [52-54]. Peptide toxins that interact with ion channels are typically made of 10-45 amino acids in invertebrates from the Cnidaria phyla and the Conidae family, namely the cone snails. In invertebrates of the Arthropoda phyla, such as the scorpions and spiders, and vertebrates of the Reptilia class, such as the snakes, most toxins are of up to 75 amino acids. All these toxins are characterized by a low molecular weight (< 10 kDa) and one to five cysteine bridges [18,55-58].

The patterns of distribution of the cysteine residues in the primary sequences generate frameworks which in turn result in particular tridimensional arrangements of α-helices, β-strands, loops, and disulfide bonds that stabilize the structure of the peptides, giving origin to the large, surprising, and complex structural diversity of toxins. The folding patterns originated by the disulfide bonds confer optimal three-dimensional interactions with its target receptor sites, as well as resistance to proteases, high temperatures, pH changes, and harsh chemicals [59,60]. A very large number of tridimensional arrangements have been recognized for invertebrate and vertebrate channel-modulating toxins. For instance, folds A, B, and C (inhibitor cystine knot ‒ICK‒ motif) are the most common arrangements in cone snail toxins [56]. Folds A and B are small structures rich in α-helices, while the ICK motifs are formed by two or three strands of β sheets with three disulfide bridges that form a cystine knot [56,60]. The ICK motif is also common in spider and scorpion toxins. Spiders also present the disulfide-directed β-hairpin (DDH) fold and scorpion toxins are rich in the cysteine-stabilized α/β (CSα/β) and cystine-stabilized helix-loop-helix (CSαα) motifs [18,55,57,58,61]. In contrast, snake toxins can form more complex, large structures, including the three-finger toxins (3FTx, enriched in β-strands), cysteine-rich secretory proteins (CRISP, one larger domain rich in α-helices, β-strands and loops connected to a smaller domain rich in α-helices) and BPTI-Kunitz-type (α-helices, β-strands and a long inhibitory loop) peptides [62-64]. Of note, CRISP, for instance, has also been reported in spiders and scorpions, and ICK and CSα/β motifs are also present in snake toxins [63,65], highlighting that most motifs and tridimensional arrangements are not exclusive of any type of venomous animal.

Remarkably, the presence of cystine bridges and the folding patterns favored by them confer toxins the ability to bind and modulate (i.e., activate or inhibit) voltage-gated ion channels with high affinity, being therefore called disulfide bonding, or bridged, peptides (DBP) [66]. This property has been used to understand the role, diversity, structure-function relationship, gating, and tissue localization of ion channels [29,67,68]. For this reason, venoms and toxins are considered gold mines of channel regulators and therapeutic agents with a lot of potential applications [69-73]. Accordingly, several venom-based molecules are successfully used as leads in basic research and drug discovery [74-76], as will be shown in the following section.

*Main sources of channel-modulating toxins*

Cnidarian and cone snails, scorpions, spiders, and snakes are the main sources of venoms enriched in mammalian voltage-gated ion channel-modulating toxins. For each of them, we present some representative toxins described around the world and then comprehensively present the toxins characterized by Central and South American research groups.

Cnidarian and cone snails: Cnidaria and Mollusca phyla include marine invertebrates particularly enriched in toxins [77,78]. Cnidaria includes five classes and more than 10,000 species of jellyfish, sea anemones, and corals, among others [78]. The genus Conus highlights within the Mollusca phylum because it includes over 900 marine species of cone snails [77]. Toxins derived from cnidarians and cone snails target many ionic and non-ionic channels and have shown interesting applications. For example, dalazatide is a potent, selective blocker of K_v_1.3, derived from the Stichodactyla toxin (ShK) of the venom of the Stichodactyla helianthus sea anemone, which is in Phase II clinical trials for the treatment of immune diseases [79]. Similarly, the potent MNT-002 antibody-ShK conjugate has served to better understand the K_v_1.3 pore-blocking dynamics at atomic resolution, further supporting ShK conjugates’ prospective use as immunomodulators [80].

The classification of the large amount of disulfide-rich toxins derived from cone snails is complex, and it has been performed according to their gene superfamily, their cysteine framework, and their pharmacological targets [56,77]. Conotoxins from the A, D, I, J, M, and O gene superfamilies commonly modulate voltage-gated ion channels. MVIIA, MVIIB, MVIIC, and MVIID, from Conus magus, target channels of the Ca_v_2 family [81]. Ziconotide (a synthetic analog of MVIIA) is now used to treat neuropathic pain [17,82]. Other conotoxins have potential as analgesics by acting on Ca^2+^ channels as well (e.g., FVIA, CVIB, GVIA, Tx6.7, MoVIA, MoVIB, TxVIA) [81,83-85]. The recently investigated Mu8.1 toxin from Conus mucronatus, which inhibits Ca_v_2.3 [47], highlights because it is about twice as large as the majority of conotoxins known to block ion channels. Conotoxins have also provided a variety of toxins specifically acting on some K_v_ channels. ViTx from Conus virgo inhibits K_v_1.1 and K_v_1.3, but not K_v_1.2 or K_v_11.1 [86], pl14a from Conus planorbis selectively blocks K_v_1.6, but not K_v_1.2, K_v_1.3, K_v_1.4, K_v_1.5, Na_v_1.2 or I_CaN_ [87], and κ−PVIIA from Conus purpurascens and κM-RIIIK from Conus radiatus inhibit Shaker channels [88,89]. Further studies demonstrated that κM-RIIIK and κM-RIIIJ from Conus radiatus inhibit K_v_1.2, but not K_v_1.3, K_v_1.4, Kv1.5, K_v_7.2 or K_v_11.1 [89,90]. Noticeably, the ability of κM-RIIIK to block K_v_1.2/K_v_7.2 heteromeric channels may confer it cardioprotective properties [90]. Despite the selectivity of many conotoxins, some others (e.g., μ-PIIIA, μ-SIIIA) have been shown to block K_v_, but also Na_v_ isoforms [91].

Only a few studies on cnidarian or cone snail venoms or toxins come from Central or South American laboratories. A Mexican-Argentinian collaboration showed that the complete venom of the Mexican cnidarian Palythoa caribaeorum blocks Ca_v_2.2 in rat superior cervical ganglion neurons. This venom is enriched in low molecular weight peptides (mostly ~2-4 kDa), however, the isolation of one or more specific toxins responsible for this effect constitutes an avenue for future research [92]. Moreover, two toxins, BcsTx1 and BcsTx2, from the sea anemone Bunodosoma caissarum from Brazil have shown the ability to block K_v_1.1, K_v_1.2, K_v_1.3, and K_v_1.6 isoforms with very high potency [49].

Although most cone snail species inhabit the African and Asian Ocean Pacific waters, many species are known to be present in the American coasts [77,93]. Mexican, Brazilian, and Cuban efforts have revealed the effect of some conotoxins from American coasts on voltage-gated ion channels. The toxin sr11a from Conus spurius blocks K_v_1.2 and K_v_1.6, but not K_v_1.3 [94]. Interestingly, PiVIIA from Conus princeps activates I_Ca_ in rat dorsal root ganglion neurons [95]. Many other conotoxins reported by Central and South American groups await electrophysiological characterization [96-98].

Scorpions: The best-studied toxins from the scorpion venoms act by binding to four groups of ion channels in excitable cells [57]: Na_v_ (NaScTx) [99], Ca_v_ (CaScTx) [100], K_v_ (KScTx - α-Ktx, β-Ktx, γ-KTx, δ-KTx, ε-Ktx, κ-KTx, and λ-KTx) [101,102], and Cl_v_ (ClScTx) [103]. Kurtoxin, isolated from Parabuthus transvaalicus inhibits N-type and L-type Ca^2+^ currents in thalamic neurons and slows down current activation kinetics, acting as a gating modifier toxin [104]. This toxin also demonstrated the modulatory capacity of Na_v_1.6 currents in the murine vas deferens myocytes. This toxin has a dual effect, increasing peak amplitude at potentials between -40 and -30 mV, and decreasing peak amplitude for potentials more positive than -10 mV [105]. Charybdotoxin from the venom of Leiurus quinquestriatus hebraeus, Agitoxin2 from Leiurus quinquestriatus hebraeus, Ev37 from Euscorpiops validus, margatoxin from Centruroides margaritatus and HelaTx1 from Heterometrus laoticus [106-111], inhibit several isoforms of the Shaker subfamily (K_v_1.x) of channels likely by occluding the pore, whereas BmPO5 from the Asian scorpion Buthus martensii binds to the K_Ca_2.2 [112]. Interestingly, venoms of the Androctonus genus harbor toxins with opposite effects on K_v_. While AmmTx3 from A. mauretanicus blocks K_v_4.2, AbTXKβ_(2-64)_ from A. australis activates K_v_7.3 and K_v_7.4 isoforms [113,114].

There are four main families of scorpions in Central and South America: Buthidae, Chactidae, Diplocentridae, and Liochelidae. Buthidae, the largest and most studied family, comprises five genera: Ananteris, Centruroides, Microtityus, Rhopalurus, and Tityus [101,115]. Mexico and Brazil, in collaboration with Hungarian and Belgian scientists, have contributed significantly to the electrophysiological characterization of venoms and toxins from the American scorpions. Notably, the first K^+^ channel-blocking peptide identified using voltage-clamp was Noxiustoxin (toxin II.11) [116], a toxin from Centruroides noxius, a scorpion of the Buthidae family, native from Mexico. This scorpion also expresses cobatoxins, which block K_v_1.1 channels [51]. Many other scorpion toxins isolated from species of America also block K_v_1.1, K_v_1.2, or K_v_1.3 isoforms, such as Pi1, Pi2 and Pi3 from Pandinus imperator [117], Tc30 and Tc32 from Tityus cambridgei [118], anuroctoxin from Anuroctonus phaiodactylus [119], Ts19 Frag-II from Tityus serrulatus [120], OcyKTx2 from Opisthacanthus cayaporum [121], ɑ-KTx 2.14 from Rhopalurus garridoi [122] and TstβKTx from Tityus stigmurus [123]. Centruroides margaritatus also expresses toxins selective against K_v_1.1, K_v_1.2, or K_v_1.3, such as Cm28 and Cm39 [124,125]. Many of these scorpion toxins have drawn attention due to their pharmacological potential [126]. In agreement with earlier reports by a Mexican group describing the presence of regulators or the ERG K^+^ channels, ergtoxins, in the Centruroides genus [127], a recent Mexico-Colombian collaboration reported a very potent (IC_50_ 3.4±0.2 nM in patch-clamp experiments), hERG1 inhibitor, the CmERG1 toxin, in the venom of Centruroides margaritatus [101]. The presence of this type of toxin could partially explain the cardiovascular toxicity of the venom of C. margaritatus in mammals [101,128]. Finally, it is worth to mention Discrepin, a toxin from Tityus discrepans, which blocks K_v_4.3 in several experimental models [129-131].

Spiders: ω-agatoxins, from the venom of the spider Agelenopsis aperta, act as a pore blocker of Ca_v_, but also as a gating inhibitor [132]. A peptide of 41 amino acids from the tarantula H. gigas blocks R-type Ca^2+^ currents [133]. DW13.3 toxin from Filistata hibernalis acts as a potent blocker of all Ca^2+^ channel currents, except for T-type currents [134]. Huwentoxin-X from Ornithoctonus huwena is a specific blocker of N-type Ca^2+^ currents in rat dorsal root ganglion neurons [135]. ω-grammotoxin-SIA, isolated from the venom of the tarantula Grammostola spatulata, is an N- and P-type Ca^2+^ currents blocker when the neurons are depolarized until +50 mV and behaves as a gating modifier with further depolarizations [136]. Effects on gating properties of channels carrying P-type currents seem to be shared by other toxins, such as Lsp-1 from Lycosa sp [137]. Some spider toxins also interact similarly with K^+^ channels, as illustrated by the effect of HpTx2 from Heteropoda venatoria as a gating modifier on the K_v_4 channels [138]. Interestingly, the venom of the Chinese tarantula Chilobrachys jingzhao has toxins (Jingzhaotoxins) with potent effects on Na_v_, but which also affect K_v_ channels [139]. Phrixotoxins (PaTx1 and PaTx2) from the tarantula Phrixotrichus auratus specifically block K_v_4.2 and K_v_4.3, something that has helped understand the isoforms underlying some I_K_ currents in ventricular cardiomyocytes of mammalians [140].

Twelve species of Pamphobeteus and many of the famous Phoneutria genus (e.g., P. depilata, P. reidyi, P. fera, and P. boliviensis) have been characterized in South America [141,142]. Researchers from Colombia have described sequences similar to Theraphotoxin-Pn1a, Theraphotoxin-Pn1b, and Theraphotoxin-Pn2a, known to affect Ca_v_, in the venom of Pamphobeteus aff. nigricolor [143]. Also using a bioinformatics approach, sequences with a high analogy to Ctenitoxins suggest that the venom of Phoneutria boliviensis may affect Ca_v_2.1, 2.2, and 2.3 [144]. Recently, a rich source of new neurotoxin peptides that may act on Na_v_ and Ca_v_ channels was found in the venom gland of Phoneutria depilata by using transcriptomic and proteomic approaches [142]. Patch-clamp experiments become thus necessary since they will confirm or reject the conclusions regarding these Pamphobeteus and Phoneutria venoms. Promising results are expected for several reasons. For instance, previous studies by Brazilian researchers have already shown that the venoms of other species of the Phoneutria genus present in South America harbor Ca_v_ peptide blockers [145,146]. Also, other Pamphobeteus spiders, such as P. verdolaga, a recently described species from the Colombian Andes [147], showed potential antibacterial and channel-interacting peptides in the transcriptomic analysis of its venom gland [148,149]. Preliminary assays using fluorescence showed that some fractions of the venom of this spider could block some Ca_v_ [150], a result recently confirmed by our group using the patch-clamp technique and several isolated toxins [151]. Moreover, researchers have found that other South American members of the Theraposidae family, such as Grammostola sp., have toxins that block Ca_v_ and K_v_ [50,136]. Besides these genera, the recent discovery of four Medionops species in a Colombian arachnological collection [152] reminds us that much work is ahead for the characterization of “old” and novel venoms potentially rich in channel-modulating toxins. 

Snakes: Besides the presence of ICK peptides, venoms from snakes are enriched in different types of toxins with effects on ion channels, such as the 3FTxs, CRISPs, and BPTI-Kunitz-type peptides [62]. Stejnihagin toxin from Trimeresurus stejnegeri, an Asian snake, and calciseptine, from Dendroaspis polylepis polylepis, an African snake, block I_CaL_ [153,154]. The dendrotoxins I and toxin K, from the latter snake, block K_v_1.1 [155]. Other dendrotoxins targeting K_v_1.1, K_v_1.2, or K_v_1.6 channels have been purified from the Dendroaspis genus as well [46,62]. Similarly, BF9 from Bungarus fasciatus blocks K_v_1.3 [156]. 

BaltCRP of the South American Bothrops alternatus inhibits K_v_1.1, K_v_1.3, and K_v_2.1 with a low affinity over 1 µM, but does not block K_v_1.2, K_v_1.4, K_v_1.5, or K_v_10.1, as reported by a Brazilian-Belgium collaboration [48]. The venoms of a variety of species of the Micrurus genus from the southern region of Brazil reversibly inhibit K_v_1.3 [157], however, specific toxins have not been brought to light yet. The snake toxins have been recognized as potential therapeutic tools [62]. For instance, the exendin-4 toxin from the lizard Gila monster (Heloderma suspectum), present in Mexico and the United States, antagonizes pancreatic I_K_, thus modulating the release of insulin. The effect of this toxin is mediated by the intracellular increase in cyclic adenosine monophosphate, in turn, produced by the exendin-4 activation of the glucagon-like peptide one receptor-mediated activation of trimeric G proteins. The indirect regulation of nanomolar concentrations of exendin-4 on I_K_ and also on I_CaL_ has been found useful as a tool for the treatment of type II diabetes and its complications [158-160].

**Figure 3:**
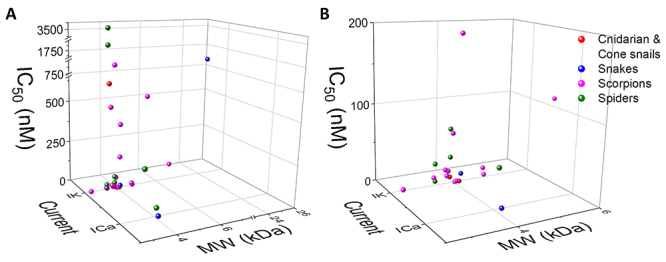
Dot plot of the molecular weight and inhibitory potency of the main toxins characterized by patch-clamp in Central and South America. **(A)** Toxins of cnidarian and cone snails (red dots), snakes (blue dots), scorpions (pink dots), and spiders (green dots) for which the molecular weight (MW) and inhibitory concentration 50 (IC_50_) have been determined in Central and South American laboratories. **(B)** Zoom to the zone of high potency and low MW to better resolve the individual toxins. One toxin may have more than one entry if it has IC_50_ values determined for several channel isoforms or current types (I_Ca,_ Ca^2+^ current; I_K_, K^+^ current). Additional file 1 shows the toxins labeled for their easier identification.

Table 1 and Figure 3 summarize the molecular weight as well as the inhibitory or activatory potency of Central and South American toxins. Most toxins (82%) have a molecular weight between 2.5 and 4.5 kDa. Almost all toxins (96.4%) were reported to block ionic currents, while only one toxin (PiVIIA) was found to activate I_Ca_ (note that this toxin was included in Table 1 but it was not represented in Figure 3). A refined analysis of the inhibitory power of the toxins that block I_Ca_ or I_K_ shows that in 40.63% of the cases, an IC_50_ between 0 and 10 nM (very potent) was measured, in 28.12% of the times the IC_50_ was over 10 and up to 100 nM (potent), the values were over 100 but lower or equal than 1,000 nM in 25.00% (moderate potency) of the cases and 6.25% of assays had an IC_50_ larger than 1,000 nM (low potency). A final observation is that there is an overrepresentation of scorpion toxins, most of which have been tested only against I_K_. Overall, the toxins characterized with patch-clamp methods in Central and South American laboratories typically weigh less than 4.5 kDa and are very potent or potent to inhibit I_Ca_ or I_K_.

**Table 1:** Summary of the toxins with effect on voltage-gated ion channels characterized by patch-clamp in Central and South America^a^

Species	Toxin	^d^ **I**_x_	^e^ **MW (KDa)**	^b^ **IC**_50_ **(nM)**				Reference
				Cnidarian and cone snails	Snakes	Scorpions	Spiders	
*Bunodosoma caissarum*	BcsTx1	^f^K_v_1.2	4.15	0.03				[49]
*Bunodosoma caissarum*	BcsTx2	K_v_1.6	3.91	7.76				[49]
*Conus spurius*	sr11a	K_v_1.6	3.65	640.00				[94]
*Conus princeps*	PiVIIA	^g^I_CaT_, I_CaN_	3.09	^c^3,000.00				[95]
*Bothrops alternatus*	BaltCRP	K_v_1.1	24.40		1,000.00			[48]
*Heloderma suspectum*	Exendin-4	K_v_	4.19		10.00			[159]
*Heloderma suspectum*	Exendin-4	^h^I_CaL_	4.19		0.10			[160]
*Centruroides noxius*	Noxiustoxin (II-11)	K_v_	4.19			390.00		[116]
*Centruroides noxius*	Cobatoxin 1	K_v_1.1	3.73			500.00		[51]
*Pandinus imperator*	Pi1	K_v_1.3	3.84			9.70		[117]
*Pandinus imperator*	Pi2	K_v_1.3	4.04			0.05		[117]
*Pandinus imperator*	Pi3	K_v_1.3	4.07			0.50		[117]
*Tityus cambridgei*	Tc30	K_v_1.3	3.87			15.90		[118]
*Tityus cambridgei*	Tc32	K_v_1.3	3.52			9.90		[118]
*Anuroctonus phaiodactylus*	Anuroctoxin	K_v_1.3	4.08			0.73		[119]
*Tityus serrulatus*	Ts19 Frag-II	K_v_1.2	5.53			544.00		[120]
*Opisthacanthus cayaporum*	OcyKTx2	K_v_1.3	3.81			18.00		[121]
*Rhopalurus garridoi*	ɑ-KTx 2.14	K_v_1.1	3.94			1,000.00		[122]
*Tityus stigmurus*	TstβKTx	K_v_1.1	6.72			96.00		[123]
*Centruroides margaritatus*	Cm38	K_v_1.2	2.82			0.96		[125]
*Centruroides margaritatus*	Cm38	K_v_1.3	2.82			1.30		[125]
*Centruroides margaritatus*	Cm39	K_v_1.2	3.98			65.00		[124]
*Centruroides margaritatus*	CmERG1	K_v_11.1	4.79			3.40		[101]
*Centruroides elegans elegans*	CeERG4	K_v_11.1	4.76			12.80		[127]
*Tityus discrepans*	Discrepin	K_v_4.3	4.17			190.00		[129]
*Phoneutria nigriventer*	Tx3-2	I_CaL_	3.55				280.00^*^	[145]
*Grammostola spatulata*	Grammotoxin	^i^I_CaP_	4.10				50.00^*^	[136]
*Grammostola iheringi*	GiTx1	K_v_4.3	3.58				2,000.00^*^	[50]
*Grammostola iheringi*	GiTx1	K_v_11.1	3.58				3,695.00	[50]
*Phrixotrichus auratus*	PaTx1	K_v_4.3	3.55				28.00	[140]
*Phrixotrichus auratus*	PaTx2	K_v_4.3	3.92				71.00	[140]
*Phrixotrichus auratus*	PaTx1	K_v_4.2	3.55				5.00	[140]
*Phrixotrichus auratus*	PaTx2	K_v_4.2	3.92				34.00	[140]

a A total of 28 different toxins have 33 entries in the table since some toxins modulate both I_Ca_ and I_K_ or more than one channel isoform. Values were either directly taken (when experimentally measured) or estimated (based on extrapolations when not experimentally determined) from the results presented in the cited references. *From those estimated, these three values are considered most likely overestimated given the poor information available in the original papers. ^b^IC_50_, inhibitory concentration 50, applies for all toxins in the table, except for PiVIIA of Conus princeps; ^c^This is the only effective concentration 50 (EC_50_) value in the table, since this toxin activates I_Ca,_ instead of inhibiting it; ^d^I_x_, ionic current; ^e^MW, molecular weight; ^f^K_v_, K^+^ channel isoform; ^g^I_CaT,_ I_CaN_, T-type Ca^2+^ current, N-type Ca^2+^ current; ^h^I_CaL_, L-type Ca^2+^ current; ^i^I_CaP_, P-type Ca^2 +^ current.

Next, we will discuss the main experimental models used to evaluate the effect of toxins on voltage-gated ion channels using electrophysiological techniques.

### Cell-free and single-cell-based models

*General concepts*

Cell-free models such as artificial bilayer lipid membranes (BLM) or single cell-based models are the most used to study the effect of toxins on ion channels. BLMs were originally developed using lipid or proteolipid extracts of neural tissue. They were soon modified to combine different types of lipids and adsorb molecules to simulate an excitable system. However, these models were mostly used to measure membrane biophysical properties (thickness, capacitance, resistance, and osmotic permeability) [161-163]. Later on, synthetic peptides or toxins inserted into phospholipids offered information on the possible features mediating the permeation pathway of ion channels [164,165]. Even when this technique is costly and time-consuming and the assembled membranes are unstable, it offers very precise information at a single-channel level and it is continuously being refined, in such a way that during the last three decades, BLM became very popular as a complementary method to study the effect of toxins or ligands on purified ion channels [166-169].

Single cells can be either derived from living animals (primary culture) or cultured cell lines. Cardiomyocytes (see section Cardiomyocytes and neurons), neurons (see section Cardiomyocytes and neurons), and T-lymphocytes are arguably the best mammalian primary culture models [119]. An alternative is using the non-mammalian oocytes from the frog Xenopus laevis [50,120,138,157]. When using cell lines, the best method is overexpressing the channels in the mammalian Human embryonic kidney (HEK) or Chinese hamster ovary (CHO) cells, or in the Sf9 insect cells [30,80,124,170]. 

Below, we will focus on cardiomyocytes and dorsal root ganglion neurons of mice as suitable primary cell models for the electrophysiological screening and evaluation of the effect of toxins on the ion channels of mammals. These two models offer advantages for many laboratories that may not be specialized in biophysical techniques: i) both are models of classical excitable cells in which a variety of macroscopic currents of sizeable amplitude can be obtained without excessive sources. Particularly, the presence of I_Ca_ makes them relevant to overcome the bias of the region in favor of evaluating the effects of toxins almost only on I_K_, as demonstrated above, ii) the preclinical studies of promising molecules require cardiotoxicity studies, iii) they can be obtained from the same animal, thus reducing costs and fulfilling ethical requirements, iv) the information they give can be used to understand many effects of poisoning by multiple venomous species or for the treatment of some of the most prevalent diseases in mammals.

*Cardiomyocytes*

Cardiomyocytes are striated muscle cells that generate contractile force in the heart [171]. A spatially defined program of ion channels is required for the cardiomyocyte to regulate contractility through the excitation-contraction coupling phenomenon [172]. Although quite irregular, a typical adult murine cardiomyocyte is cylinder-like shaped, with a length of about 120-150 μm and a diameter of around 20-40 μm. In our laboratory, we typically found cardiomyocytes’s capacitances between 105 and 160 pF (n=100). Cardiomyocytes have two separate nuclei, a highly organized array of myofilament proteins, and an extensive membranal T‐tubule network with a high density of ion channels, particularly of Ca_v_ and K_v_ [173,174].

In ventricular cardiomyocytes, Ca_v_1.2 is the most abundant isoform. It is the molecular entity responsible for the characteristic I_CaL_ found during phase two of the cardiac AP, which is activated at potentials more positive than -40 mV and reaches its peak amplitude between 0 and +10 mV [20,175]. The inactivation is biexponential, with a fast component described by a 𝛕_f_ between 12 and 16 ms and a slow component with 𝛕_s_ between 133 and 577 ms [174,176,177]. 

The presence of several K_v_ isoforms (K_v_1.1, K_v_1.2, K_v_1.4, K_v_1.5, K_v_1.7, K_v_2.1, K_v_3.1, K_v_4.2, K_v_4.3, K_v_7.1, K_v_11.1) underlies the different K^+^ currents measured in the heart (mainly the transient outward ‒I_to_‒ and the delayed rectifiers ‒I_Kur_, I_Kr_, I_Ks_‒), and their differential distribution and degree of expression across the myocardium are responsible for the differences in shape and duration of the atrial and ventricular AP [20,23,32,178-181]. I_to_ is rapidly activated and contributes to phase one of the AP. Subtypes of I_to_ currents can be separated into fast (K_v_4.2, K_v_4.3) or slow (K_v_1.4, K_v_1.7) inactivation kinetics currents (𝛕 values between 25-80 ms for I_tof_ and 80-200 ms for I_tos_) [23]. I_K_ (K_v_1.1, K_v_1.2, K_v_2.1, K_v_1.5, K_v_3.1, K_v_7.1, K_v_11.1) contribute to the phases three and four of the AP and include the subtypes of currents: I_Kur_, I_Kr,_ and I_Ks_. They typically activate at potentials more positive than -30 mV. I_Kur_ and I_Kr_ rapidly activate but become inactive with differential kinetics. On the contrary, I_Ks_ activate slowly but inactivate rapidly.

*Dorsal root ganglion neurons*

Spinal ganglia are located along the dorsal roots of the paraspinal nerves: cervical, thoracic, lumbar, and sacra. Inside these ganglia reside pseudo-unipolar neurons, which transport sensitive information from the skin and internal organs. These dorsal root ganglion (DRG) neurons are typically rounded, with a central nucleus, abundant Golgi, and endoplasmic reticulum [182,183]. The DRG neurons are protected by other small cells (satellite cells), usually adhered to the neuronal soma [184]. Molecular markers such as neurofilament 200 and β-III-tubulin are abundant in the DRG neurons but absent in the support cells [185-187]. For this reason, they are markers used to differentiate cell populations in the DRG.

DRG neuronal populations can be classified according to cell soma diameter o peripheral conduction velocity (CV) as small (< 20 μm), intermediate (21-40 μm), and large (> 40 μm). The small cells (fibers C) are devoid of myelin and have low CV (0.7-2.3 m/s). The intermediate cells (fibers A-δ) are moderately myelinated and thus conduct at somewhat faster velocities (3-15 m/s). The larger cells (fibers Aβ y Aα) are robustly myelinated and reach very fast conduction velocities (20-80 m/s) [184,188]. Obtained from mice or rats, this cellular model is suitable for the study of the effect of toxins on Ca_v_ [95,189], K_v_ [50,95,190], Na_v_ [50,191,192], and other transporters [193], in patch-clamp experiments. These cells have membrane capacitances between 44.3±14.4 pF (fibers C) and 70.0±27.6 pF (fibers Aβ and Aα) and a typical resting membrane potential of -60.0 mV [188]. These neurons differentially express P/Q, N, R, and T-type Ca^2+^ currents [12]. I_K_ is also diverse in these types of neurons, where K_v_1, K_v_3, and K_v_4 are the mainly expressed subfamilies [194,195]. 

## Methodological aspects

### Cardiomyocytes and neurons

*Isolation of cardiomyocytes*

**Figure 4:**
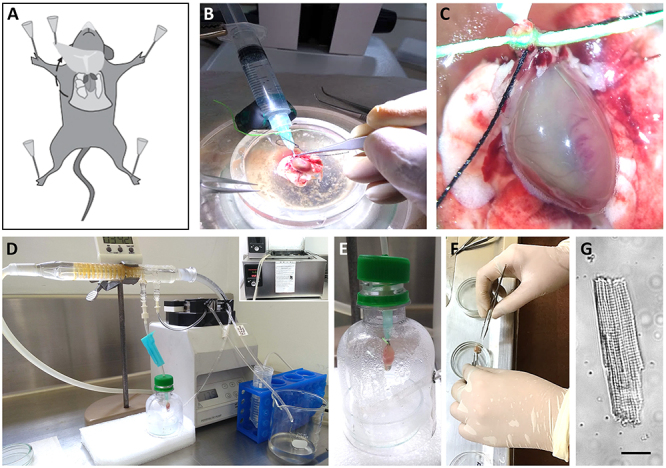
Schematic representation of murine cardiomyocytes isolation. **(A)** Procedure to pin the mouse´s limbs and gain access to the thoracic cavity. **(B, C)** Once the heart is removed, it should be rapidly mounted on the cannula, which is a shortened, tip-polished needle. Proper magnification allows the tying of the aorta to the cannula, with the help of two silk strands. Blood is then washed out by gently pushing the syringe-stored anticoagulant-supplemented Tyrode solution through the aorta. **(D)** The cannulated heart is transferred to the homemade Langendorff apparatus. It has a source of heated water (inset), a perfusion system driven by a pump, and a hoses network to ensure the solutions with different compositions reach the heart or are recycled. **(E)** A tight control of the temperature can be achieved by keeping the heart inside a closed chamber. **(F)** Once the heart has been enzymatically digested and looks pale, it is dismounted and minced with the help of forceps and scissors. **(G)** This procedure renders hundreds of isolated, living cardiomyocytes. Calibration bar: 50 µm.

To obtain a single-cell suspension of cardiomyocytes, the heart needs to be digested. However, cardiac cells are firmly adhered to each other by the intercalated disks and the extracellular matrix. Moreover, cardiomyocytes are susceptible to hypoxia, physiological deterioration, mechanical perturbations, nutrient availability, pH, temperature changes, ionic fluctuations, and enzymatic digestion [196]. A common procedure that guarantees a good quality of cardiomyocytes is the Langendorff technique [197]. The main principle of this method is to perfuse the heart with enzyme-containing solutions in a retrograde manner. The retrograde flow shuts the leaflets of the aortic valve so that the perfusion solution cannot enter the left ventricle, being thus evacuated into the coronary arteries [198] (Figure 4).

The procedure starts with the removal of the heart from the thoracic cavity of the animal model in no more than 2 minutes (Figure 4 A ). Adult mice of the Swiss Webster or C57BL/6, of 20-26 g, render good results. It is better to leave as much of the ascending aorta as possible, including the lungs, to facilitate cannulation. All solutions must be filtered with 0.22 µm nitrocellulose filters. The tissue is rapidly immersed twice in cold, Ca^2+^-free Tyrode solution (in mM: NaCl 135, KCl 5.4, MgCl_2_ 1, Glucose 10, HEPES 10, NaH_2_PO_4_ 0.33, pH 7.3) with enoxaparin (1 mg/mL) (Figure 4 B ). 

The aorta is then cannulated under a stereoscope, the lungs are removed, and the heart is transferred to the Langendorff system (Figure 4 C  and 4D). At this stage, the heart is perfused at a flow rate of 4.5 mL/min with Ca^2+^-free Tyrode’s solution, bubbled with 95% O_2_ and 5% CO_2_, and supplemented with 1 mg/mL collagenase (type 2; Worthington, Lakewood, NJ) and 0.1 mg/mL protease (type XIV; Sigma, St. Louis, MO); always kept at 37°C for ~20 min, until the heart becomes pale and soft to the touch (Figure 4 E ). Only the ventricles are transferred to a Petri dish containing warm Ca^2+^-free Tyrode’s solution and triturated into small pieces with forceps (Figure 4 F ). After a gentle agitation, dozens of isolated, intact cardiomyocytes appear in the solution (Figure 4 G ). Finally, small amounts of Ca^2+^ are slowly restored to the solution.

*Isolation of dorsal root ganglion neurons*

**Figure 5:**
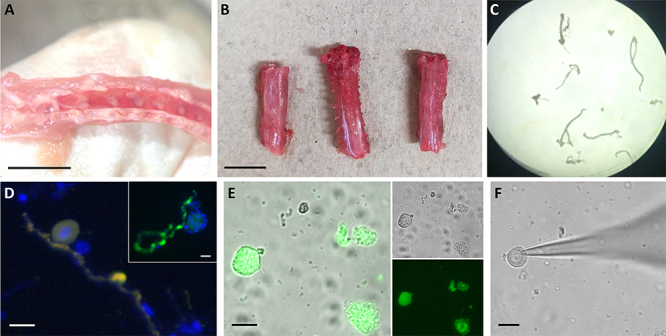
Schematic representation of murine dorsal root ganglion neuron isolation. **(A)** The ganglia look like dots inside the spine. **(B, C)** Dividing the spine into its cervical, thoracic, and lumbar parts makes ganglia removal easier. **(D)** After the enzymatic treatment, the identity of the isolated neurons can be characterized by demonstrating the positivity of the cells to neuron markers by fluorescence microscopy. In the large panel, the yellow color indicates the merge of the fluorescence channels imaging antibodies-labeled neurofilament 200 (originally labeled in green) and β-III-tubulin (originally labeled in red). The inset shows a different neuron only labeled for neurofilament 200 (green). Blue corresponds to nuclei, as labeled with Hoechst. Since each neuron has only one nucleus, other nuclei reflect the presence of small support cells still attached to some parts of the neurons. **(E)** The cells were loaded with the fluorescent Ca^2+^ dye Mag-Fluo-4. The large panel shows the merge of the bright field (upper inset) and the fluorescence field (lower inset). The appearance of the Ca^2+^ fluorescence (green) helps distinguish viable (leftmost cell) from non-viable (right, lowermost cell) cells because of the severe Ca^2+^ compartmentalization in the latter. **(F)** Demonstrates the feasibility of the path-clamp experiments in these neurons. The shadow coming from the right of the image corresponds to the patch clamp micropipette, which looks attached to the cell membrane at the leftmost part of the image. Calibration bars: 1 cm (A, B), 25 µm (D-F).

After removing the cardiopulmonary apparatus and the viscera, two longitudinal cuts are made on either side of the vertebral column and one transversal cut below vertebrae L6. The longissimus muscles are removed, and the vertebral column can then be divided into cervical, thoracic, and lumbar sections and kept in cold Ca^2+^-free Tyrode’s solution. New cuts in the sagittal plane along the vertebral canal will help expose the spinal cord and the associated right and left DRG (Figure 5 A , 5B, and 5C). This method renders ~35 DRG per mouse [187,192,199].

The DRG neurons are then incubated in 3 mg/mL collagenase type 2 in Ca^2+^-free Tyrode’s solution at 37.5°C for ~60 min. After washing out the enzyme, the DRG neurons are again incubated in 2.5 mg/mL Trypsin in Ca^2+^-free Tyrode’s solution at 37.5°C for ~12 min. The DRG neurons can then be dissociated. A gentle centrifugation at room temperature helps concentrate the neurons, whose nature can be verified as shown in Figures 5 D  through 5F.

### I_Ca_ and I_K_ studies

**Figure 6:**
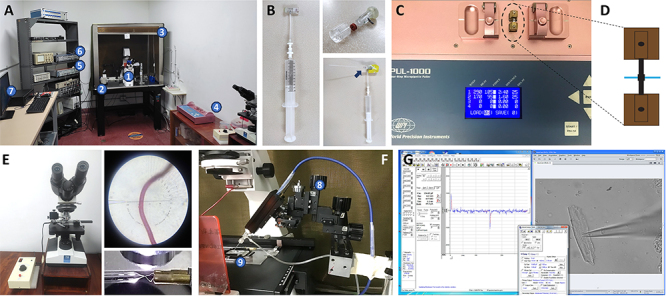
Electrophysiology setup for a patch-clamp experiment. **(A)** Electrophysiology setup. An inverted microscope (1) equipped with large magnification and a digital camera for adequate visualization of the working field (see panel **F** below) is mounted on an anti-vibration table (2) to reduce mechanical noise and is enclosed by a Faraday cage (3) to reduce electromagnetic noise. A nearby station to fabricate micropipettes includes a puller (4) and a microforge. On the left, the rack with the amplifier (5) and the digitizer (6), is close to the acquisition and processing hardware and software (7). **(B)** Home-made holder for cleaning the glass capillaries. This simple gadget is ensembled by tightly joining the embolus of a syringe to a hollow adapter (upper inset) through a thin silicone tube of 2-3 cm in length. Once the capillary is connected to the female port (blue arrow), air can be rapidly flushed to remove any dust from the inside of the capillary. **(C)** Typical protocol used to fabricate micropipettes by pulling the glass capillaries. The heating element, zoomed in **(D)**, heats the capillary (drawn in blue), allowing it to be stretched and split to form two micropipettes. **(E)** Each micropipette´s tip is forged with the help of a small, incandescent filament (red in the upper inset, as seen from the eyepiece of the microscope shown in **E**). A lateral view of the procedure is shown in the bottom inset (the micropipette coming from the left, and the filament from the right). **(F)** The forged micropipette is filled with the internal solution and mounted in the electrophysiology headstage equipped with an Ag/AgCl electrode, thus becoming a microelectrode. A micromanipulator (8) is then used to bring the recording microelectrode close to the isolated cells plated in the bath solution in the experimental chamber (9). A reference electrode put in the external solution closes the circuit. **(G)** Typical software configuration used in our laboratory. On the left, the interface of the recording software shows the conditions representative of a successful whole-cell experiment with a cardiomyocyte. After a gigaseal (> 1 GΩ) is formed, the cell membrane is ruptured, as effectively shown by the capacitive currents (peaks upwards and downwards) in the blue line and the numerical values of the parameters: Cm (membrane capacitance), Rm (membrane resistance), Ra (access resistance), Tau (time constant of decay of the capacitive peaks). On the right, the camera live image shows the seal between the microelectrode, coming from the right, and the cardiomyocyte. In the middle is the interface of the electrophysiology amplifier, in which the voltage-clamp mode is selected. After this moment, the ionic currents can be recorded.

A setup for patch-clamp studies is shown in Figure 6 A . Cleaning the glass capillaries can be considered the most important preparation step for patch-clamp experiments [200]. This can be achieved by soaking them in ultra-pure water for two hours and then fully heat-drying them in an oven. Furthermore, we always apply positive pressure inside the capillaries using a homemade holder connected to a syringe (Figure 6 B ). Micropipettes must be fabricated immediately with the clean capillaries, using the common two-stage process: pulling a capillary and thus heat polishing the 3-5 µm pipette tip. Schott glass capillaries (outside diameter 1.65 mm, inside diameter 1.20 mm, Schott 8250 composition -today’s equivalent of Corning’s old 7052 glass-) give good results (Figure 6 C, 6D and 6E).

Table 2 shows selected solutions used to perform patch-clamp experiments aimed at measuring I_Ca_ and I_K_ in different cellular models. While one solution type is inside the micropipette, the other solution type baths the cells. All solutions should be filtered through a cellulose filter.

**Table 2:** Selected solutions to perform patch-clamp experiments in different cellular models^a^.

Cellular model, current [reference]	I_Ca_						I_K_							
	Cardiomyocytes, I_CaL_ [151,201]		HEK293T, I_CaT_ [47]		Dorsal root ganglion neurons, I_Ca_ [95]		Cardiomyocytes, I_K_ [151]		CHO, K_v_11.1 [101]		Dorsal root ganglion neurons, I_K_ [95]		Peripheral blood mononuclear cells, K_v_1.3 [125]	
	Pipette (mM)	Bath (mM)	Pipette (mM)	Bath (mM)	Pipette (mM)	Bath (mM)	Pipette (mM)	Bath (mM)	Pipette (mM)	Bath (mM)	Pipette (mM)	Bath (mM)	Pipette (mM)	Bath (mM)
NaCl			5	110				135	10	95				145
KCl							140	5.4		40	50	10		5
KF											40		140	
K^+^ aspartate									130					
K^+^ gluconate			140											
CaCl_2_		1		10	0.1	2.5		1		2	0.1	1.8	1	2.5
MgCl_2_		1	2	1			2	1	2	2		1.2	2	1
MgATP	5				2		4				2			
^b^HEPES	11	10	10	10	15	10	10	10	10	10	5	10	10	10
^b^EGTA	10		5		10		5		10		10		11	
D-glucose		10		10				10		5				5.5
^b^Chol-Cl											60	130		
CsCl	125	6		5	130	5								
CdCl								^c^0.3				0.3		
^b^TEA-Cl		140		30	10	130								
^b^TTX		0.1						0.1						
NaH_2_PO_4_								0.33						
Na_2_GTP					1						1			
^b^4-AP						10								
pH	7.1 (adj. with CsOH)	7.4 (adj. with CsOH)	7.2 (adj. with KOH)	7.35 (adj. with TEA-OH)	7.2	7.4	7.2 (adj. with KOH)	7.2 (adj. with NaOH)	7.3 (adj. with NaOH)	7.3 (adj. with NaOH)	7.2	7.4	7.22	7.35
^d^Osmolarity (mOsm/L)	~280	~315	~295	~340	~310	~298	~308	~308	~308	~295	~320	~300	~310	~315

a Solutions used to measure I_Ca_ (Ca^2+^ currents) and I_K_ (K^+^ currents) in different cellular models in different laboratories. ^b^HEPES: 2-[4-(2-hydroxyethyl)piperazin-1-yl]ethanesulfonic acid; EGTA: 2-[2-[2-[2-[bis(carboxymethyl)amino]ethoxy]ethoxy]ethyl-(carboxymethyl)amino]acetic acid; Chol-Cl: Choline chloride; TEA-Cl: Tetraethylammonium chloride; TTX: tetrodotoxin; 4-AP: 4-Aminopyridine. ^c^Nifedipine 10 µM can also be used. ^d^These theoretical values overestimate by 5-10% of the expected real values because van´t Hoff corrections were not applied. HEK: human embryonic kidney cell line; CHO: Chinese hamster ovary cell line; I_CaL_: L-type Ca^2+^ current; I_CaT_: T-type Ca^2+^ current. K_v_, K^+^ channel isoform.

Once the pipette is filled and mounted on the headstage of the patch-clamp setup, its tip is immersed into the bath solution, placed in the middle of the visual field, and checked. If the tip is clean and smooth and the pipette resistance is between 1.5 and 3 MΩ, it can be displaced until reaching close contact with the cellular membrane (Figure 6 F  and 6G). As long as the electrical values are satisfactory (seal is > 1 GΩ), the whole-cell configuration can be established by applying a small suction with a syringe. Complementarily, in devices equipped with a Zap circuit, a large (~ 1 V), brief (~ 0.1-10 ms) voltage pulse can be applied through the electrode, just by clicking on the Zap function of the interface program, thus increasing the probability of gaining access to the cytoplasm of the cell. Compensation of the Rs is usually adjusted up to 50% to minimize voltage control errors, and the P/4 protocol can be used to subtract leakage currents. The automatic fast and slow capacitance and whole-cell compensation functions are activated to reduce capacitances. Samples should be recorded at 10-20 kHz and can be low-pass filtered (Figure 6 G ).

**Figure 7:**
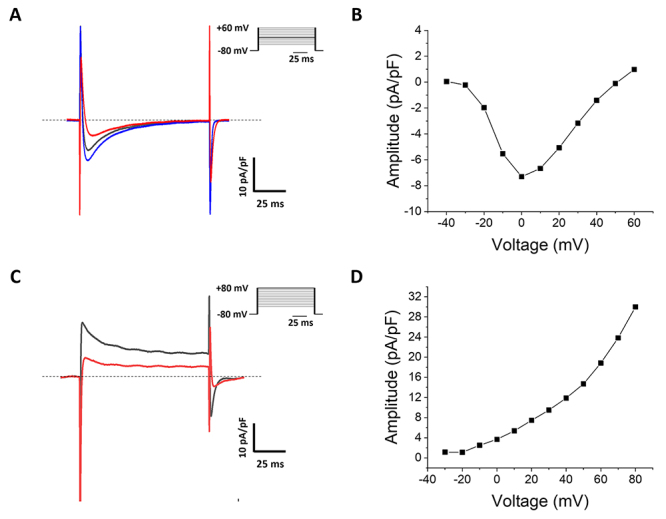
Typical currents and electrophysiological characterization of a peptide toxin from the P. verdolaga spider in isolated mouse cardiomyocytes. **(A)** I_CaL_ control (black), activated by 10 µM isoproterenol (blue) and blocked by 1 µM vrdg177 (red), even in the presence of isoproterenol. Only the currents obtained at maximum activation are shown (voltage step highlighted in black in the voltage-clamp protocol). **(B)** An I_CaL_ current-voltage plot of a different cardiomyocyte obtained according to the steps of the voltage-clamp protocol shown in A. The maximum current activation is observed at 0 mV. **(C)** I_K_ control (black) and partially blocked by tetraethylammonium (red). Only the currents obtained at maximum activation are shown (voltage step highlighted in black in the voltage-clamp protocol). **(D)** An I_K_ current-voltage plot of a different cardiomyocyte obtained according to the steps of the voltage-clamp protocol shown in C. All analyzed currents had membrane seal resistances over 1 GΩ, with access resistances lower than 10 MΩ. Experiments were performed at room temperature.

The typical kinetics of I_CaL_ and I_K_ obtained in isolated cardiomyocytes are shown in Figure 7. The kinetics of the electrophysiological signals and their response to pharmacological modulators help experimentally confirm the identity and quality of the currents. For instance, under the stimulation protocols presented in Figure 7, and the appropriate solutions described in Table 2, I_CaL_ are inward currents, plotted as downward deflections (Figure 7 A ), with a maximum activation seen between 0 and +10 mV (Figure 7 B ) [175]. Classically, nifedipine (5-15 µM) blocks I_CaL_ while isoproterenol (1-10 µM) potentiates it (Figure 7 A ) [15,175,176]. In this preparation, the underlying molecular entity of I_CaL_ is Ca_v_1.2 [20]. On the other hand, the protocol and solutions used for I_K_ in ventricular cardiomyocytes elicit macroscopic outward currents, graphed as upward deflections (Figure 7 C ), with maximum activation at +80 mV (Figure 7 D ). TEA (at high concentrations) and phrixotoxin-2 (100-300 nM) block this I_K_ [140]. This current mostly reflects the activation of K_v_4.2, K_v_4.3, K_v_1.4, and K_v_1.7 in this experimental model [20].

### Study case

A new species of Tarantula was recently discovered in the Colombian Andes, named Pamphobeteus verdolaga [147]. This discovery represents a valuable opportunity to study novel toxins with potential use in physiology, pharmacology, and toxicology. A transcriptomic analysis of the venom gland of this species revealed polypeptide sequences which were confirmed to have antimicrobial properties [148,149]. Since some of the predicted toxins were short, bridged peptides, we hypothesized about a potential effect on ion channels. Several of these peptides were locally synthesized and screened for effects on ion channels. As a result, a few of them showed to be channel blockers [151]. Figure 7 shows currents (A and C) and current-voltage relationship plots (B and D) for I_CaL_ and I_K_ obtained in cardiomyocytes. vrdg177 (vrdg stands for verdolaga and 177 comes from the transcriptomic analyses) at a concentration of 1 µM was able to block more than 50% of I_CaL_ in the illustrative recordings of Figure 7 A . This peptide has 18 amino acids, a molecular weight of 2.3 kDa, a charge of +5, an isoelectric point of 9.8, water solubility higher than 4 mg/mL, and two disulfide bridges. As shown above (Figure 3), concentrations between 0.5 and 1 µM are within a good ballpark to start a screening of the potential effect of toxins on ion channels under electrophysiological approaches [202-205]. These results show that an initial screening identified spider toxins blocking I_Ca_, as opposed to many other American toxins demonstrated to block I_K_. This study case implies high novelty because spider venoms and toxins are barely studied with patch-clamp techniques and because it reflects a successful example of the comprehensive study of a toxin by implementing a multidisciplinary approach within one South American country. In this case, a national collaboration of several researchers allowed the description of the species and its taxonomic classification, then the local bioinformatic analyses of the peptide sequences permitted to demonstrate the potential of the new toxins, which led to the use of biochemical techniques to synthesize the peptides, whose success permitted the biophysical experiments which finally contributed to their electrophysiological characterization.

## Conclusion

As this review did not search Chinese databases (e.g., Chinese Medical Current Content (CMCC), China National Knowledge Infrastructure (CNKI)), relevant information about toxins of Asian and African origin is likely underrepresented [206]. Also, toxins with an effect on voltage-gated Na^+^ channels were excluded. This precluded us from deeply discussing very interesting and particular profiles of toxins such as OD-1. This toxin from the Iranian scorpion Odonthobuthus doriae specifically activates Na^+^ currents, a property proven useful by a Taiwanese group to standardize a novel model of seizures and excitotoxicity [207], with multiple applications in biomedical research. However, the text highlights very interesting profiles of American toxins and keeps focused on the main aspects of its evaluation by patch-clamp, so the above limitations do not affect the main aims and conclusions of the review.

The main perspective implies that considerable effort should be made to better characterize the wealth of old and new molecules purified by many Latin American and Brazilian groups studying natural products. For instance, many venoms seem to have effects on ion channels, but specific channel-interacting toxins have not been isolated yet [92,157]. Marine sources are understudied in the region as channel modulators, even when the access to the sea is vast. Also, in earlier papers, the effects of many toxins were tested against a limited number of cellular models and channel isoforms, which hinders information about the selectivity of those toxins. The standardization of models for I_Ca_ studies is highly encouraged since a clear bias in favor of I_K_ analyses has thus arisen. This requires strengthening the electrophysiological capabilities in our region, which can be achieved by standardizing the use of the patch-clamp technique in different models. A network that brings together natural products research groups with biophysical research groups may also help to that aim. Our laboratory will be glad to be part of this network and collaborate with other laboratories in the region. This is relevant for the bioprospection of natural products in a continent rich in biodiversity and toxins that potently inhibit ion channels. Toxinological applications include the understanding of the venom complexity of a large number of species. Toxicology applications refer to the study of the mechanisms of action of toxins in potential prey and humans. Biotechnological applications comprise the exploration of potential applications of toxins as research, agricultural, or biomedical tools. Moreover, better collaborations can be done with research groups around the world.

In conclusion, the patch-clamp highlights as an electrophysiological technique relevant to the study of the potential effects of peptide toxins on voltage-gated ion channels. An initial evaluation can be implemented in cardiomyocytes and DRG neurons. Establishing these techniques in several laboratories across the region will strengthen the research capabilities in many fields, with copious potential applications. 

## Abbreviations

AP: action potential; BLM: bilayer lipid membranes; BPTI-Kunitz: bovine pancreatic trypsin inhibitor-Kunitz polypeptides; Ca_v_: voltage-gated Ca^2+^ channels; CHO: Chinese hamster ovary; C_m_: membrane capacitance; CRISP: cysteine-rich secretory proteins; CV: conduction velocity; DBP: disulfide bonding peptides; DDH: disulfide-directed β-hairpin; DHP: dihydropyridines; DPPX: dipeptidyl-aminopeptidase-like protein 6; DRG: dorsal root ganglion; EGTA: 2-[2-[2-[2-[bis(carboxymethyl)amino]ethoxy]ethoxy]ethyl-(carboxymethyl)amino]acetic acid; ERG: ether-a-go-go related gene; 3FTx: three-finger toxins; 𝑔: conductance; HEK: human embryonic kidney; HEPES: 2-[4-(2-hydroxyethyl)piperazin-1-yl]ethanesulfonic acid; I_C_: capacitive current; I_Ca_: Ca^2+^ current; ICK: inhibitor cystine knot motif; I_K_: K^+^ current; I_t_: total current; I_x_: ionic current; KchAP: K_v_ channel-associated protein; KChIP: K_v_ channel-interacting protein; KCNE: K^+^ voltage-gated channel subfamily E regulatory subunit; KCNQ or KvLQT: K^+^ voltage-gated channel subfamily Q member or voltage-gated K^+^ channel isoform associated to long QT syndrome; K_v_: voltage-gated K^+^ channels; PD: pore domain; Rs: series resistance; SF: selectivity filter; SNR: signal-to-noise ratio; TEA: tetraethylammonium; TTX: tetrodotoxin; V_m_: membrane potential; VSD: voltage sensing domain.

## Data Availability

Not applicable.

## Supplementary material

The following online material is available for this article:

Additional file 1 Dot plot of the molecular weight and inhibitory potency of the main toxins characterized by patch-clamp in Central and South America. All toxins were labelled with their names for their easier identification.
