# Supplementary material for: Electrophysiological evaluation of the effect of peptide toxins on voltage-gated ion channels: a scoping review on theoretical and methodological aspects with focus on the Central and South American experience
Source: J Venom Anim Toxins Incl Trop Dis. 2024 Sep 2;30:e20230048. doi: 10.1590/1678-9199-JVATITD-2023-0048 (PMC11389830; doi:10.1590/1678-9199-JVATITD-2023-0048)
Supplement: Additional file 1 [file 1678-9199-jvatitd-30-e20230048-s1.pdf]

J Venom Anim Toxins incl Trop Dis
https://doi.org/10.1590/1678-9199-JVATITD-2023-0048

Supplementary Material to "Electrophysiological evaluation of the effect of peptide toxins on voltage-gated ion channels: a scoping review on theoretical and methodological aspects with focus on the Central and South American experience"

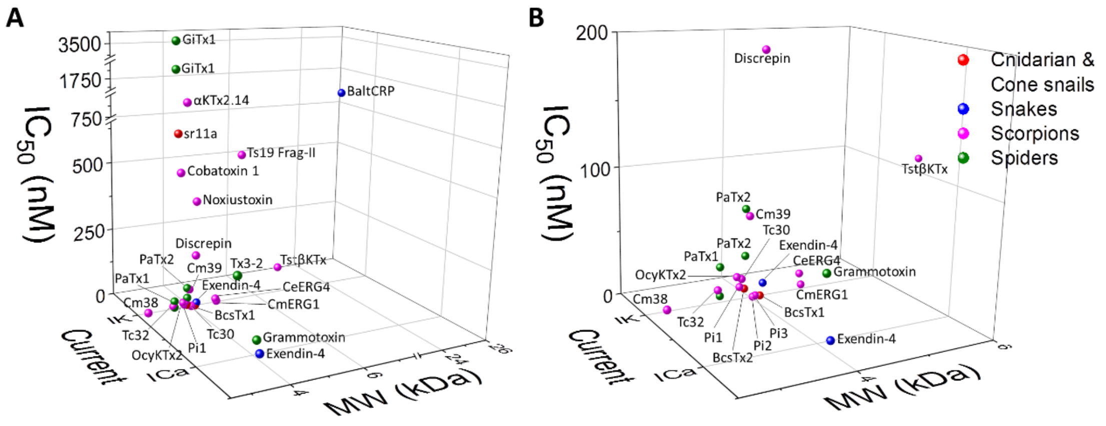

Additional file 1. Dot plot of the molecular weight and inhibitory potency of the main toxins characterized by patch-clamp in Central and South America. All toxins were labelled with their names for their easier identification.
